# Primary adult unilateral thalamic pilocytic astrocytoma with von Recklinghausen's disease mimicking lymphoma: A case report

**DOI:** 10.1016/j.radcr.2022.03.094

**Published:** 2022-04-18

**Authors:** Mehdi Borni, Firas Jarraya, Ines Cherif, Mouna Zghal, Naouraz Gouiaa, Mohamed Zaher Boudawara

**Affiliations:** a Department of Neurosurgery, UHC Habib Bourguiba, Street Majida Boulila, Sfax 3029, Tunisia; b Department of Neurosurgery, Valenciennes Hospital Center, Valenciennes, France; c Laboratory of Pathology, UHC Habib Bourguiba, Sfax, Tunisia

**Keywords:** Thalamic pilocytic astrocytoma, Lymphoma, MRI, Surgery

## Abstract

Thalamic astrocytomas are rare central nervous system tumors that account for 1%-1.5% of all brain tumors. Their Clinical features depend on anatomical involvement. For these tumors, gross total resection is so difficult due to their deep location and also the infiltration of the optic pathway or brain stem. Unilateral adult thalamic locations are rarely described in the literature. Their radiological features often suggest lymphoma. The authors report here a new case of a primary unilateral thalamic pilocytic astrocytoma mimicking lymphoma diagnosed after a stereotactic core biopsy in a 62-year-old male patient with von Recklinghausen's disease and which is responsible for Dejerine–Roussy syndrome. The authors will proceed with a comprehensive review of literature regarding this rare entity.

## Introduction

Pilocytic astrocytoma (PA) is a tumor originally described by Harvey Cushing in 1931 [1]. PAs are benign brain tumors (grade I of the World Health organization classification and spatial grading of type I) [2], [3], [4]. These are the most common brain tumors found in children [4]. They are found ubiquitously in the central nervous system, with a predilection for optic pathways, diencephalon, third ventricle, and especially the posterior fossa. PA of the diencephalon include the thalamic, hypothalamic and those of the basal ganglia. They have been little described and for them the literature remains scarce. Unilateral adult thalamic locations are rarely described in the literature. Their clinical features depend on anatomical involvement and their radiological features often suggest lymphoma. For these tumors, gross total resection is so difficult due to their deep location and also the infiltration of the optic pathway or brain stem.

The authors report here a case of a primary unilateral thalamic pilocytic astrocytoma mimicking lymphoma in a 62-year-old male with von Recklinghausen's disease which is responsible for Dejerine–Roussy syndrome. The patient underwent a stereotactic core biopsy using the Lars Leksell with uneventful course. The authors will proceed with a comprehensive review of literature regarding this rare entity.

## Case report

A 62-year-old male patient with medical history of von Recklinghausen's disease since childhood not well investigated and acute pancreatitis 8 months before admission without any particular surgical history who was admitted to our neurosurgery department for progressive onset, 2 months before admission, of lower and upper left limbs burning and tingling sensations, widely varying in degree of severity during the day accompanied by hypersensitivity in form of dysaesthesia associated to a moderate gait disorder.

**Fig. 1:**
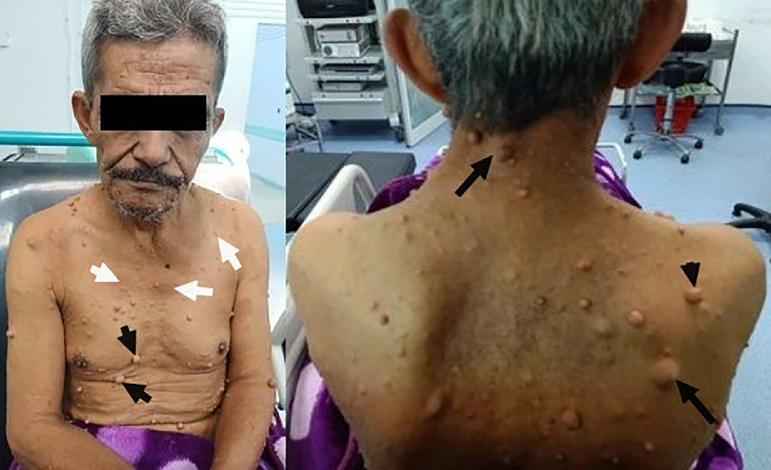
Clinical photography of the patient showing several soft tissue cutaneous nodules (neurofibroma; black arrows), covering the whole body including the head, neck, chest, limbs, dorsal region with multiple hyperpigmented macules (Café-au-lait spots; white arrows).

Upon neurological examination we discovered hemiparesis and hyperesthesia of his left body with anosognosia and somatoparaphrenia defining the Dejerine–Roussy syndrome. The rest of the body examination revealed several soft tissue cutaneous nodules (neurofibroma), covering the whole body including the head, neck, chest, limbs, dorsal region and even the scalp with multiple hyperpigmented macules (Café-au-lait spots) without freckling in the underarms (axillary) or groin (inguinal) regions defining the von Recklinghausen's disease (Neurofibromatosis type I) (Fig. 1). Deep ophtalmic examination revealed chronic anterior uveitis without abnormal clumps of pigment on the colored portion of the eye (Lisch nodules) or papillary oedema.

**Fig. 2:**
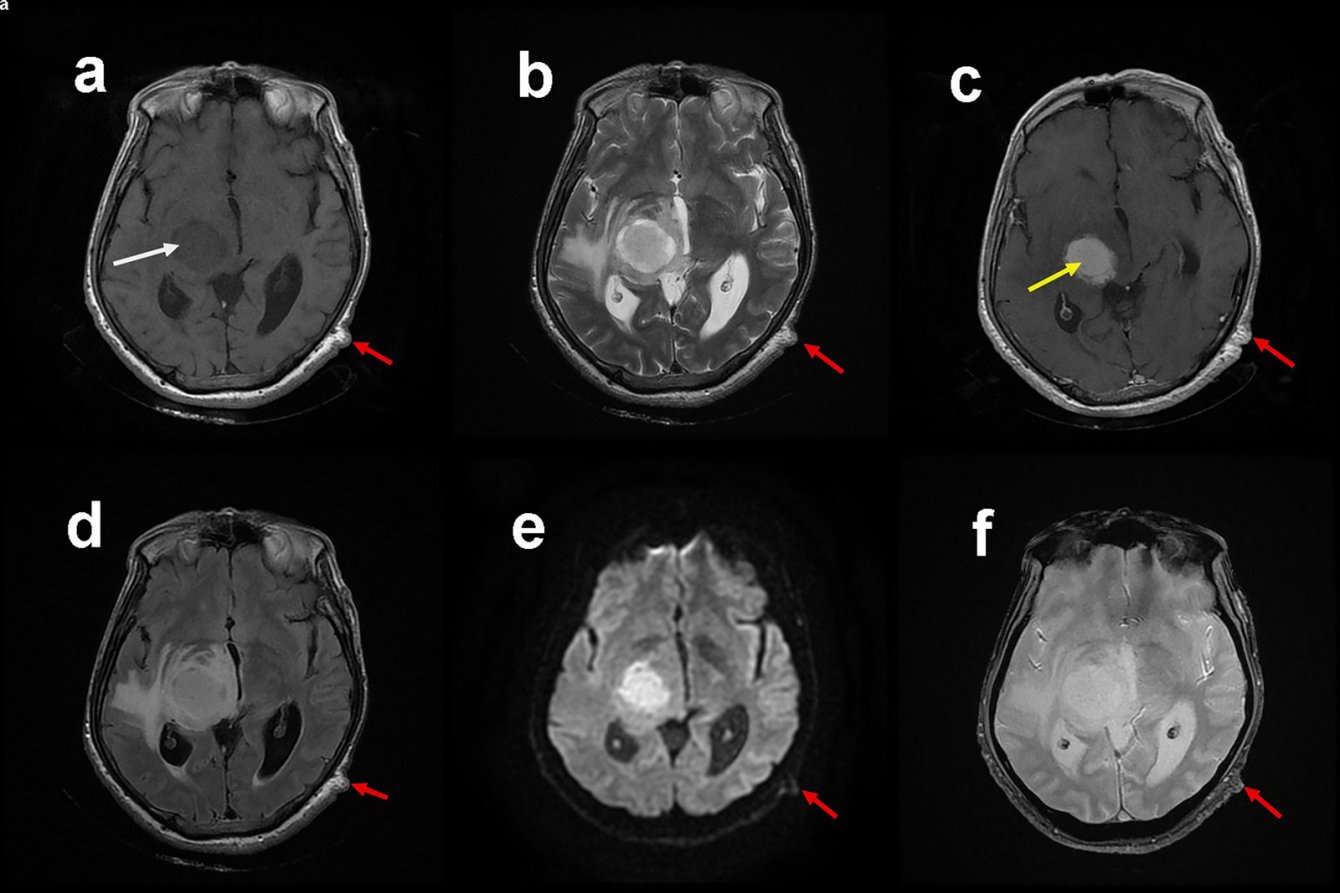
Axial MR images obtained at the time of clinical onset showing a right capsular and thalamic mass (white arrow), measuring 23 × 30 × 31 mm in long axes, hypointense on T1 weighted images (A) with heterogeneous hypersignal on T2 sequences (B), and which is intensely and homogeneously enhanced after injection of Gadolinium chelates having the aspect of a snowball (C; yellow arrow). Note the surrounded significant perilesional edema on T2 Fluid attenuated inversion recovery sequences (FLAIR) (D) exerting a mass effect on the ipsilateral ventricle, midline and the third ventricle. Diffusion-weighted imaging (DWI) revealed a moderate hypersignal (E). There was no evidence of bleeding on the gradient echo sequence (F). Note the neurofibroma of the scalp in the different sequences (red arrow). (Color version of figure is available online.)

Cerebral magnetic resonance imaging (MRI) (Fig. 2) showed a right capsular and thalamic mass, measuring 23 × 30 × 31 mm in long axes, hypointense on T1 weighted images with heterogeneous hypersignal on T2sequences, and which is intensely and homogeneously enhanced after injection of Gadolinium chelates having the aspect of a snowball. This process partially invades the ipsilateral cerebral peduncle and is surrounded by significant perilesional edema reaching the brainstem and which exerts a mass effect on the ipsilateral ventricle, midline and the third ventricle on T2 Fluid attenuated inversion recovery sequences. Diffusion-weighted imaging revealed a moderate hypersignal with reduced signal on Apparent Diffusion Coefficient mapping. There was no evidence of bleeding on the gradient echo sequence. On magnetic resonance spectroscopy, biochemical and metabolic environment of the tissue were characterized by a significant preponderant lipid peak, small choline peak, drop in N-Acetyl Aspartate, and a myoinositol peak. Perfusion-weighted imaging sequence shows a supershift above the baseline without hyperperfusion. All these features were in favor of cerebral lymphoma in an immunocompetent patient. In front of this hypothesis, high-dose steroid therapy was not administered to our patient as it may causes rapid shrinkage in size of the cerebral lymphoma, with loss of distinctive histological findings.

The biological assessment of white and red blood cell showed no abnormalities as well as in platelets and C-reactive protein test. Serology for Hepatitis B, C, and human immunodeficiency virus were negative.

Our patient underwent a stereotactic core biopsy using the Lars Leksell Stereotactic system with scannographic tracking to allow the brain mapping in a 3-dimensional coordinate system and select the appropriate target coordinates for guiding the biopsy needle. The procedure was performed under local and regional scalp nerve block.

**Fig. 3:**
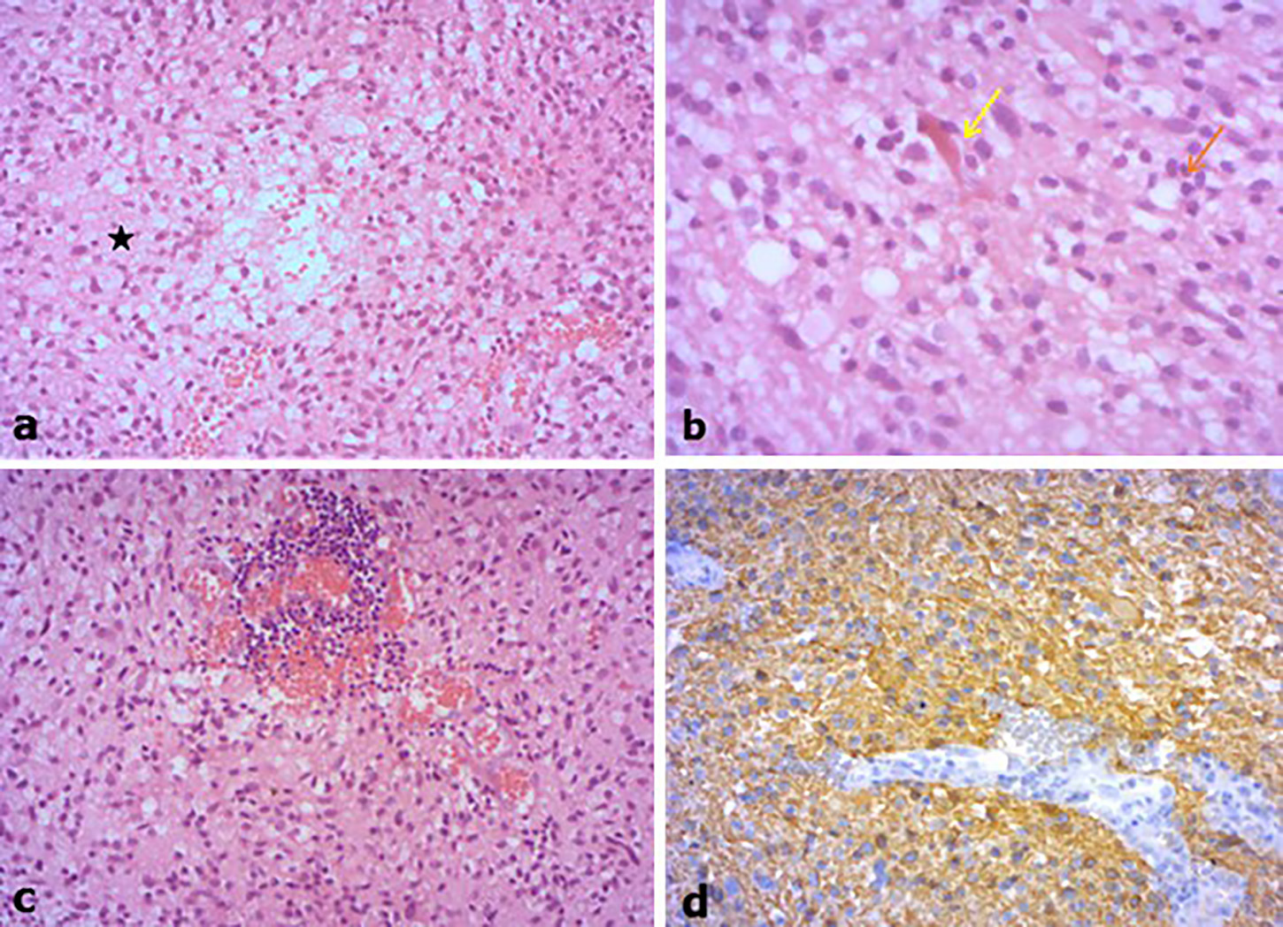
(A) Glial proliferation with biphasic pattern (black asterisk) alternating with compact area of moderate cellularity and loose architectural pattern (HEX100). (B) Round to bipolar neoplastic cells with bland or hyperchromatic nuclei and microcystic features (red arrow); note the Rosenthal fibers (yellow arrow) (HEX 400). (C) Perivascular lymphocytes infiltration (HEX200). (D) Strong expression of GFAP (X200). (Color version of figure is available online.)

Histological examination revealed a glial proliferation with moderate cellularity, sitting in a fibrillary background. The tumor has a biphasic pattern alternating with compact and loose areas (Fig. 3A). Neoplastic cells were round to elongate with bland or hyperchromatic nuclei. Mitotic figures were exceeding rare (1 mitose/High power field). Occasional microcystic changes and Rosenthal fibers were seen (Fig. 3B). The tumor was highly vascularized with perivascular lymphocytes infiltration (Fig. 3C). Necrosis and glomeruoid vascular proliferation were absent. Immunohistochemically, the tumor cells showed strong positive stain for GFAP (Fig. 3D). Finally, the diagnosis of PA was established.

Postoperatively, no complications or worsening of his neurological condition were observed.

## Discussion

PA is a tumor originally described by Harvey Cushing in 1931 [1]. PAs are benign brain tumors (grade I of the World Health organization classification and spatial grading of type I) [2], [3], [4]. PA of the diencephalon include the thalamic, hypothalamic and those of the basal ganglia. They have been little described in the literature previously. Unilateral adult thalamic locations are rarely described in the literature. Thalamic forms are readily responsible for clinical signs related to obstruction of the cerebrospinal fluid flow pathways (intracranial hypertension) or neurological deficits by compression of the internal capsule (frequent hemiparesis) [5]. In our case hemiparesis was also found as well as homolateral hemihyperaesthesia with anosognosia and somatoparaphrenia defining the Dejerine–Roussy syndrome. Time between the first clinical signs and diagnosis is up to 6 months in the literature [6]. In our case we found a time period of 2 months. Generally, the clinical signs are slowly progressive (indolent, slow growing tumor); they can, more rarely, be fast, even “explosive.” The associated hypothalamic forms can also manifest as diencephalic syndrome (Russel syndrome) [7,8]. It is mostly found in infants and children, with a male predominance. Imaging tells us first about the tumor's topography. In thalamic forms, the tumor may invade the third ventricle or the lateral ventricle, and possibly present as predominantly intraventricular, making it difficult to precisely identify the tumor's origin site [4]. The characteristics of PA in MRI are: hypointense on T1-weighted sequence, hypersignal on T2-weighted sequence and in proton density and, in the vast majority of cases, intense contrast enhancement after Gadolinium injection [9,10] suggesting lymphoma. In our case, we found completely superimposable radiological features which led us to evoke the diagnosis of cerebral lymphoma.

It is currently not possible to predict the degree of aggressiveness of PA on radiological data alone; several criteria were studied: location, morphology, size, presence of calcifications, and intensity of contrast enhancement [11]. The tumor's course in PA is still unpredictable. These tumors may remain stable, decrease in size or even regress completely, either after partial surgery (including biopsy), or spontaneously for some authors. Lepto-meningeal dissemination (LMD) is rare [12], its incidence is poorly understood, between 0.7% and 12% of cases depending on the series [12], [13], [14]. Spinal cord MRI may play a role in the more frequent detection of this LMD [14]. Indeed, the predilection areas of LMD are especially the peri-medullary subarachnoid spaces (lumbosacral region and cauda equina). The tumor location most at risk for LMD is the diencephalon, and in particular the thalamus [12,^13^,14]. We did not observe any LMD for our patient and the full spine MRI was not performed as the tumor. Histologically, PA is a tumor with low to moderate cellularity, having a biphasic structure with variable proportions [4]: Compact area consisted of bipolar piloid cells associated often with Rosenthal fibers, and stellate cells with round bland nuclei, micro-cysts changes and eosinophilic granular bodies. These features were also present in the description of our specimens. Less frequently, pseudo-oligodendroglial pattern may be found. Classic histological parameters (cytonuclear atypia, mitosis, necrosis, microvascular proliferation), so useful for the grading of other astrocytomas, do not necessarily have the same unfavorable prognostic significance when it comes to PA [3]. On the other hand, the mitotic index seems to be a better prognostic marker [15] and may make it possible to decide whether or not to prescribe adjuvant treatment.

Therapeutically, when anatomical conditions allow, PA may and should be removed entirely [16], [17], [18]. No additional treatment is then offered [17,19]. Thalamic tumors have long been considered (and still are by many authors) as inaccessible to radical surgery [20], [21], [22], [23]. Factors limiting their excision are linked to many anatomical conditions: proximity of the internal capsule and the subthalamic nuclei for the ventro-lateral aspect of the thalamus. Since the early 1990s, the possibility of complete excision of thalamic tumors has been considered again, using neuronavigation means and with low morbidity [24], [25], [26], [27].This attitude of maximum resection, subject to low morbidity, is, of course, very useful for these grade I tumors. Stereotactic core biopsy may be performed to remove a mass effect in rare cystic forms. This biopsy may sum up the treatment if the tumor appears inextricable as it was done for our patient. Regarding the surgical approach for thalamic locations, we do not share the opinion of Gillet and Villarejo who recommend a trans-temporal approach [8,27] because of the risk of vascular injury which appears to be greater. Although our preference is for the Stereotactic core biopsy, the choice of the first route is however discussed depending on the anatomical conditions of the tumor.

Chemotherapy is an important part of the treatment of diencephalic and hypothalamic-chiasmatic PAs. Many protocols exist to treat diencephalic (thalamic and hypothalamic) PA, especially described by North American or Japanese teams, most often on small cohorts of patients. The purpose is to achieve tumor control and to avoid or delay radiotherapy (RT). The best indication seems to be progressing PA, for which no reasonable surgical intervention can be considered, or children with a worsening of general condition to undergo surgery and under aged for RT [13,^14^,^28^,29].

Some authors no longer use RT as first-line (in the absence of signs of malignant degeneration), or second-line after incomplete resection [27]. RT, in the context of thalamic and/or hypothalamic AP, can be offered as a complement to other therapies. It may also be considered in case of incomplete resection when there is a symptomatic tumor residue or when patient follow-up seems uncertain. Regarding our case, we preferred to opt for stereotactic biopsy and radiotherapy to minimize the risks and preserve the patient's motor function.

PA generally has an excellent prognosis, but recurrent thalamic and hypothalamic forms may lead to the patient's death, most often after a long course marked by multiple recurrences [17,18]. The prognosis may be hampered by a LMD. With regard to supratentorial PAs, for which very little data exists, Shaw et al. found a 5- and 10-year survival rate of 85% and 79%, respectively, which gives them the best prognosis for low-grade supratentorial tumors [30]. Tumor forms with bithalamic involvement have a very reserved prognosis [25,30]. In Reardon's series [30], all of the children died within 2.5 years of diagnosis. These tumors most often correspond to grade II-IV lesions; it is therefore quite exceptional to diagnose PA there.

## Conclusion

PA is an original, very atypical tumor that abounds in paradoxical features and has a good prognosis. Very little data is available with regard to thalamic and hypothalamic PA. Complete excision, if ever possible, remains the treatment of choice. The high chemo- and radiosensitivity of these tumors is a notion to be balanced, on a case-by-case basis, with the possible morbidity of a surgical procedure. Moreover, the histological grading system used for glial tumors is poorly adapted and proves disappointing for PA. The mitotic index seems to us to be an important data to collect, the only objective factor for these tumors with such unpredictable evolution, and can participate in the decision to prescribe adjuvant treatments.

## Patient consent

Written informed consent was obtained from the patient for publication of this case report and any accompanying images.
